# Comparative analysis of postoperative outcomes following hysterectomy versus sacrocolpopexy: Insights from global federated health research network^☆^

**DOI:** 10.1016/j.eurox.2025.100433

**Published:** 2025-10-30

**Authors:** Muhammed A.M. Hammad, MacKinnly T. Knoerzer, Gamal M. Ghoniem

**Affiliations:** Department of Urology, University of California, Irvine, 3800 W. Chapman Ave. Ste. 7200, Orange, CA 92868, United States

**Keywords:** Hysterectomy, Pelvic organ prolapse, Postoperative complications, Sacrocolpopexy

## Abstract

**Objective:**

To compare the risk of opioid use, antimicrobial utilization, and postoperative complications, including paralytic ileus, between patients undergoing abdominal hysterectomy and sacrocolpopexy.

**Methods:**

Using a global federated health research network, we performed a retrospective cohort analysis. After 1:1 propensity score matching for age, BMI, race, ethnicity, diabetes, and hypertension, 993 patients were included per group. Kaplan-Meier survival analysis assessed antimicrobial and opioid use, resistance, and opioid abuse or dependence.

**Results:**

Mean follow-up was significantly longer in the sacrocolpopexy group (7.67 ± 4.66 years) than in the hysterectomy group (3.13 ± 4.23 years). Hysterectomy patients experienced a higher mean number of postoperative complications although these results were not statistically significant (p = 0.0688). Postoperative antimicrobial use was significantly higher in the hysterectomy group (25.9 %) than sacrocolpopexy (14.1 %) (HR 3.84, 95 % CI: 2.99–4.93; p < 0.0001). Opioid use was also more frequent in hysterectomy patients (44.4 % vs. 15.6 %; HR 5.68, 95 % CI: 4.69–6.88; p < 0.0001), with a higher incidence of abuse/dependence (6.04 % vs. 2.02 %; HR 6.91, 95 % CI: 4.07–11.73). No significant difference was observed in antimicrobial resistance (p = 0.4409). Paralytic ileus was rare and not significantly different between groups.

**Conclusion:**

Hysterectomy was associated with greater risk of postoperative opioid use, opioid abuse/dependence, and antimicrobial use. These findings support individualized surgical planning and proactive postoperative management.

## Highlights

• Greater risk of postoperative antimicrobial use with abdominal hysterectomy.
• Abdominal hysterectomy is associated with greater risk of postoperative opioid use.
• Higher risk of opioid dependence/abuse with abdominal hysterectomy.

## Introduction

Sacrocolpopexy and hysterectomy are surgical interventions indicated for female pelvic pathology. The utility of sacrocolpopexy is primarily in pelvic organ prolapse as the gold standard for apical compartment prolapse repair [1]. Conversely, the indications for hysterectomy are variable and include endometriosis, leiomyoma, fibroids, and pelvic organ prolapse among other conditions [2]. Despite these differences, sacrocolpopexy and hysterectomy are performed in the same anatomical region and involve similar surgical approaches, including open abdominal and minimally invasive techniques [1], [2], [3]. These operations also share overlapping postoperative risks.

Sacrocolpopexy and hysterectomy are independently associated with various postoperative complications including surgical site infections, postoperative pain, and gastrointestinal disturbances [1], [2]. These complications impact quality of life, and postoperative recovery, and have economic implications [4]. These complications also underscore additional challenges with opioid analgesics and antibiotics prescribed postoperatively for pain control and infection, respectively. Notably, opioid use introduces concerns for prolonged utilization whereas antibiotic use raises the concern for development of antimicrobial resistance. Moreover, augmented and prolonged use of these medications is associated with adverse events which, collectively, create challenges for the healthcare system [5], [6].

While there are previous studies that independently evaluate the postoperative outcomes of sacrocolpopexy and hysterectomy, data that directly compares the postoperative profiles of these operations is largely absent in the current literature. Understanding outcomes and the differences between these two operations is critical for patient counseling and guiding expectations, perioperative planning, and risk stratification. Therefore, the objective of our study is to compare postoperative opioid and antibiotic use as well as complications, with a focus on paralytic ileus, associated with hysterectomy and sacrocolpopexy. Using a real-time global federated health database, we aim to clarify comparative postoperative risks and provide further insight to guide informed surgical decision-making.

## Methods

We performed a retrospective cohort study utilizing data from the TriNetX Research Network, a federated platform aggregating de-identified electronic health records from participating healthcare institutions globally. Adult female patients (aged ≥18 years) who underwent hysterectomy without concurrent sacrocolpopexy (Cohort 1) or sacrocolpopexy without hysterectomy (Cohort 2) were identified using standardized ICD-10-PCS, SNOMED, and Current Procedural Terminology (CPT) codes within the TriNetX Research Network. These codes specify the performed procedure but do not differentiate between open, laparoscopic, or robotic approaches. Because our objective was to compare the procedures themselves rather than the surgical route, both cohorts included all approaches collectively. This design ensured comprehensive and representative assessment of real-world postoperative outcomes across a global network.Patients undergoing both procedures or vaginal hysterectomy were excluded. In total, 306,363 patients were identified in the abdominal hysterectomy cohort and 1124 in the sacrocolpopexy cohort.

To minimize baseline differences between the groups, we performed 1:1 propensity score matching (PSM) using a greedy nearest-neighbor algorithm with a caliper of 0.1 pooled standard deviations. Patients were matched on the following covariates: age at surgery, diabetes mellitus (ICD-10 E08–E13), hypertensive diseases (I10–I1A), body mass index (BMI), race, and ethnicity. After matching, each cohort included 993 patients, with post-matching standardized mean differences (SMD) < 0.1 for all matched variables, indicating acceptable balance.

The primary outcomes of interest were overall postoperative complications, opioid utilization, and antimicrobial utilization. Overall postoperative complications were defined using a composite of CPT and ICD-10-CM codes that reflect hemorrhagic, infectious, and thromboembolic events. (Table 3) Opioid utilization was defined as either a systemic opioid prescription or a diagnosis of opioid use recorded postoperatively. Diagnoses were based on ICD-10-CM codes from the F11.9x series, including F11.90 (unspecified, uncomplicated). Antimicrobial utilization was defined as a prescription for any systemic antibacterial or antifungal agent postoperatively. Topical, ophthalmic, and intravaginal preparations were excluded from the analysis. Secondary outcomes included opioid abuse or dependence and antimicrobial resistance.

Kaplan-Meier (KM) survival analyses were performed to estimate time to key postoperative outcomes, including first recorded use of antimicrobials, antimicrobial resistance, opioid prescription, and opioid abuse or dependence. For each outcome, patients with diagnosis prior to the index surgery were excluded from analysis. Time zero was defined as the date of surgery, and patients were followed until the first occurrence of the event, loss to follow-up, or end of the observation period. The KM estimates extended up to approximately 7000 days (∼19.2 years), reflecting the maximum follow-up duration available in the TriNetX dataset. Although most events occurred within the first few years postoperatively, this extended timeline allowed for detection of delayed complications and evaluation of long-term event-free survival. Survival differences between surgical cohorts were assessed using the log-rank test, and hazard ratios (HRs) with 95 % confidence intervals (CIs) were estimated using Cox proportional hazards models.

All statistical analyses, including propensity score matching and comparative risk analyses, were conducted within the TriNetX platform using built-in analytic tools for risk ratio estimation and p-value generation. As the study used only de-identified aggregate data, it was deemed exempt from institutional review board (IRB) oversight.

## Results

993 matched patients were well balanced across all covariates used in the matching algorithm, with SMD’s below 0.01 for age, diabetes, hypertension, BMI, race, and ethnicity (*p* > 0.9 for all except BMI) (Table 1, Table 2, Table 3).

**Table 1:** Baseline characteristics: pre and post propensity score matching cohorts’ summary.

Characteristic	Hysterectomy (Pre-Match)	Sacrocolpopexy (Pre-Match)	p-value (Pre-Match)	SMD (Pre-Match)	Hysterectomy (Post-Match)	Sacrocolpopexy (Post-Match)	p-value (Post-Match)	SMD (Post-Match)
Age (mean± SD)	51.7 ± 13.0	61.72 ± 10.53	< 0.001	0.85	61.69 ± 10.50	61.70 ± 10.50	0.98	0.001
Diabetes mellitus (%)	9.43 %	2.72 %	< 0.001	0.28	2.62 %	2.72 %	0.89	0.0062
Hypertension (%)	23.99 %	10.26 %	< 0.001	0.37	10.27 %	10.27 %	1	0
BMI (mean± SD)	30.85 ± 8.13	25.5 ± 0.71	< 0.001	0.63	22.89 ± 0.05	25.5 ± 0.71	< 0.001	5.22
White (%)	49.85 %	59.96 %	< 0.001	0.2	59.92 %	59.92 %	1	0
Female genital prolapse (%)	8.42 %	30.28 %	< 0.001	0.58	10.07 %	30.31 %	< 0.001	0.521
Opioid analgesic use (%)	44.23 %	3.22 %	< 0.001	1.1	19.03 %	3.22 %	< 0.001	0.519
Antimicrobial use (%)	44.76 %	2.52 %	< 0.001	1.15	17.12 %	2.52 %	< 0.001	0.506
C-reactive protein (mg/L, mean± SD)	19.74 ± 44.56	52.27 ± 72.03	0.0047	0.54	19.38 ± 42.08	52.27 ± 72.03	0.0133	0.558
Leukocytes (x10^9/L, mean± SD)	55.60 ± 455.09	6.79 ± 1.98	0.3256	0.15	7.47 ± 2.71	6.79 ± 1.98	0.0283	0.288
Neutrophils (x10^9/L, mean± SD)	173.1 ± 976.1	19.0 ± 136.5	0.1479	0.22	30.27 ± 385.5	19.0 ± 136.5	0.793	0.039
Lymphocytes (x10^9/L, mean± SD)	51.70 ± 318.0	30.47 ± 260.99	0.5406	0.07	9.31 ± 109.1	30.47 ± 260.99	0.3058	0.106
Erythrocyte sedimentation rate (mm/hr, mean± SD)	22.44 ± 21.65	22.0 ± 0.0	0.9482	0.03	31.48 ± 26.77	22.0 ± 0.0	0.2757	0.501
Serum protein (g/dL, mean± SD)	7.11 ± 1.03	6.78 ± 0.89	0.0665	0.34	7.04 ± 1.01	6.78 ± 0.89	0.1716	0.271

Pre and post-match characteristics are outlined. Parameters for matching were age, diabetes, hypertension, BMI, race, and ethnicity. BMI was significantly higher among sacrocolpopexy patients after matching.

**Table 2:** Female genital prolapse incidence across the 993 matched patients in both groups.

**ICD-10 Code**	**Diagnosis**	**Hysterectomy n (%)**	**Sacrocolpopexy n (%)**	**p-value**	**SMD**
N81	Female genital prolapse	100 (10.07 %)	301 (30.31 %)	< 0.0001	0.521075
N81.4	Uterovaginal prolapse, unspecified	39 (3.93 %)	34 (3.42 %)	0.550997	0.026762
N81.1	Cystocele	36 (3.63 %)	111 (11.18 %)	< 0.0001	0.291546
N81.2	Incomplete uterovaginal prolapse	36 (3.63 %)	92 (9.27 %)	< 0.0001	0.231192
N81.6	Rectocele	21 (2.12 %)	106 (10.68 %)	< 0.0001	0.35535
N81.3	Complete uterovaginal prolapse	17 (1.71 %)	32 (3.22 %)	0.030023	0.097493
N81.9	Female genital prolapse, unspecified	13 (1.31 %)	10 (1.01 %)	0.52922	0.02824
N81.5	Vaginal enterocele	10 (1.01 %)	27 (2.72 %)	0.004785	0.126866

Multiple varieties of POP demonstrated higher preoperative incidence among patients undergoing sacrocolpopexy compared to hysterectomy. Appropriately, POP was more frequently encountered as the indication for surgical intervention with sacrocolpopexy.

**Table 3:** Compared outcomes and their associated codes.

**Outcome**	**Associated Codes**
**Overall Postoperative Complications**	CPT: 1008663; ICD−10-CM: T81.4, N99.82B, I82.40, I82.409, I82.419, I82.429, I82.439, I82.489, I82.481, I82.499, I82.621, I82.622, I82.723, I82.724, I82.729, I82.89, I82.91, I82.99, T81.719 A, T26, I78
**Opioid Utilization**	VA: CN101; CPT: 08632, 08633; ICD−10-CM: F11.9x series (e.g., F11.90, F11.93, F11.94, F11.98)
**Opioid Abuse or Dependence**	ICD−10-CM: F11.1x and F11.2x series (e.g., F11.10, F11.120, F11.159, F11.20, F11.220, F11.23, F11.282); Z79.891
**Antimicrobial Utilization**	VA: AM000 (includes AM114, AM200, AM250, AM300, AM400, AM700, AM900)
**Antimicrobial Resistance**	ICD−10-CM: Z16

Postoperative outcomes for all matched patients were analyzed within TriNetX. Search terms spanned five categories relevant to our study with multiple terms included per assessed outcome to ensure comprehensive analysis.

Mean age was 61.7 ± 10.5 years in both groups. Median follow-up duration was substantially longer for patients undergoing sacrocolpopexy at 7.95 years (IQR: 6.0 years) compared to 1.03 years (IQR: 5.0 years) in the hysterectomy group. Similarly, mean follow-up time was longer in the sacrocolpopexy cohort (7.67 ± 4.66 years) than in the hysterectomy cohort (3.13 ± 4.23 years). Female genital prolapse (N81) remained significantly more prevalent in the sacrocolpopexy group, with 301 patients (30.13 %) compared to 100 patients (10.07 %) in the hysterectomy group (p < 0.001; SMD = 0.5211). (Supplementary Table 1) Exposure to opioid analgesics (CN101) was higher in the hysterectomy group (19.033 % vs. 3.223 %; *p* < 0.0001; SMD = 0.5194), as was use of antimicrobials (AM000) (17.12 % vs. 2.518 %; *p* < 0.0001; SMD = 0.5062).

Outcomes were then further analyzed using the associated codes. (Supplementary Table 2) In the matched cohort analysis, a total of 24 patients from the hysterectomy group and 10 patients from the sacrocolpopexy group were excluded due to having a documented complication prior to the defined postoperative time window. After exclusions, hysterectomy patients experienced a higher mean number of overall complications (2.38 ± 4.56) compared to sacrocolpopexy patients (1.26 ± 0.74), though this difference was not statistically significant (p = 0.0688). The incidence of paralytic ileus was low in both groups, with ≤ 10 events reported per cohort. Among affected patients, the mean number of ileus-related events was higher in the hysterectomy group (2.5 ± 2.1) compared to the sacrocolpopexy group (1.0 ± 0), though this difference was not statistically significant (p = 0.4226).

**Fig. 1:**
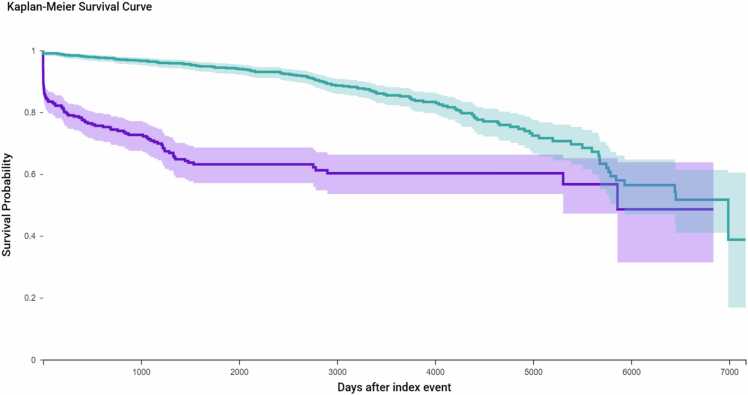
Kaplan-Meier estimates of time to first antimicrobial prescription following surgery for the matched cohort. Kaplan-Meier survival curves were generated to evaluate the time to first postoperative antimicrobial prescription in the matched cohort. After excluding patients with antimicrobial use prior to surgery (n = 506 hysterectomy; n = 54 sacrocolpopexy), 487 hysterectomy and 939 sacrocolpopexy patients remained. During follow-up, 126 hysterectomy patients (25.9 %) and 132 sacrocolpopexy patients (14.1 %) received a new antimicrobial prescription. Median time to event was longer in the sacrocolpopexy group (6990 days vs. 5860 days). The difference in antimicrobial-free survival was statistically significant (log-rank p < 0.0001), with a hazard ratio of 3.835 (95 % CI: 2.985–4.928), indicating a significantly higher rate of postoperative antimicrobial use in the hysterectomy cohort.

KM survival analysis was used to assess the time to first documented antimicrobial prescription following surgery. (Fig. 1) To ensure accurate outcome attribution, 506 patients in the hysterectomy cohort and 54 patients in the sacrocolpopexy cohort were excluded due to having antimicrobial prescriptions prior to the index procedure. This left 487 hysterectomy patients and 939 sacrocolpopexy patients for analysis. During follow-up, 126 patients (25.9 %) in the hysterectomy group and 132 patients (14.1 %) in the sacrocolpopexy group received antimicrobial prescriptions. The estimated antimicrobial-free survival probabilities at the end of the study period were 48.6 % for the hysterectomy group and 38.8 % for the sacrocolpopexy group. This difference was statistically significant (log-rank p < 0.0001), with a hazard ratio (HR) of 3.84 (95 % CI: 2.99–4.93), indicating a substantially higher risk of postoperative antimicrobial use among hysterectomy patients. A separate KM analysis was conducted to assess time to first diagnosis of antimicrobial resistance (ICD-10 Z16) following surgery. After excluding patients with preexisting resistance (n = 0 in hysterectomy; n = 10 in sacrocolpopexy), the remaining cohort included 993 hysterectomy and 991 sacrocolpopexy patients. During follow-up, 11 hysterectomy patients (1.11 %) and 17 sacrocolpopexy patients (1.72 %) developed new resistance diagnoses. Despite this numerical difference, there was no statistically significant difference in antimicrobial resistance risk between groups (log-rank p = 0.4409; HR = 1.35, 95 % CI: 0.63–2.93).

**Fig. 2:**
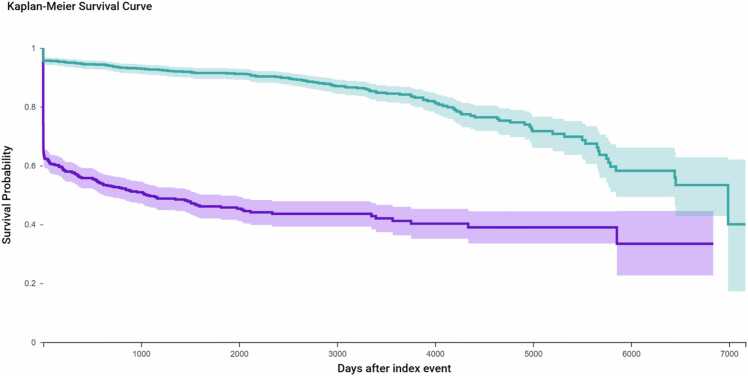
Kaplan-Meier survival curves comparing time to first postoperative opioid prescription in the matched cohort. Time to first opioid prescription following surgery was assessed using Kaplan-Meier analysis in the matched cohort (n = 993 per group). During follow-up, 441 patients (44.4 %) in the hysterectomy cohort and 155 patients (15.6 %) in the sacrocolpopexy cohort received a postoperative opioid prescription. The median time to opioid use was shorter in the hysterectomy group (1058 days) compared to the sacrocolpopexy group (6990 days). At the end of the time window, opioid-free survival was significantly lower in the hysterectomy cohort (33.4 %) compared to sacrocolpopexy (40.0 %), with a hazard ratio of 5.678 (95 % CI: 4.686–6.881; log-rank p < 0.0001).

KM survival analysis was conducted to evaluate time to first recorded postoperative opioid prescription between patients undergoing hysterectomy and those undergoing sacrocolpopexy (Fig. 2). Both cohorts included 993 patients after matching. During follow-up, 441 (44.4 %) of the hysterectomy cohort and 155 (15.6 %) of the sacrocolpopexy cohort received at least one postoperative opioid prescription. Opioid-free survival probabilities at the end of the time window were 33.4 % for the hysterectomy group and 40.0 % for the sacrocolpopexy group. The difference in opioid use was statistically significant (log-rank p < 0.0001), and patients in the hysterectomy cohort were significantly more likely to receive postoperative opioids, with a hazard ratio (HR) of 5.68 (95 % CI: 4.69–6.88).

**Fig. 3:**
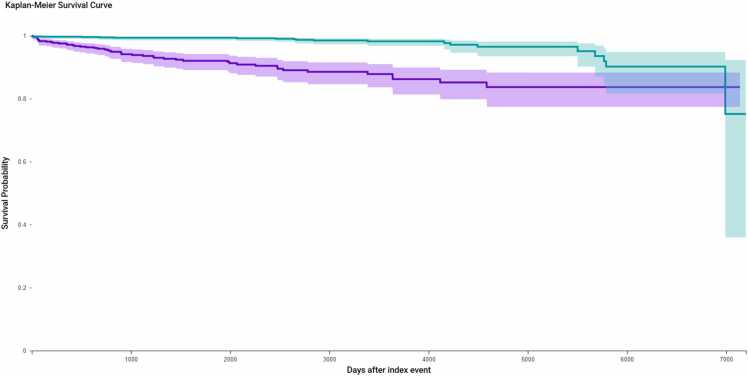
Kaplan-Meier analysis of postoperative opioid abuse or dependence in matched cohorts undergoing hysterectomy versus sacrocolpopexy. Patients with preoperative diagnoses were excluded (n = 99 hysterectomy; n = 10 sacrocolpopexy). Among the remaining patients (n = 894 hysterectomy; n = 990 sacrocolpopexy), time to first postoperative diagnosis of opioid abuse or dependence was assessed using Kaplan-Meier survival analysis. During follow-up, 54 patients (6.0 %) in the hysterectomy cohort and 20 patients (2.0 %) in the sacrocolpopexy cohort developed a new diagnosis. Event-free survival was significantly lower in the sacrocolpopexy group (log-rank p = 0.0009), with a hazard ratio of 6.91 (95 % CI).

To further assess the risk of opioid-related complications, a separate KM analysis was performed for ICD-coded opioid abuse or dependence (Fig. 3). Patients with preexisting diagnoses were excluded (n = 99 from the hysterectomy group; n = 10 from the sacrocolpopexy group), resulting in analytic cohorts of 894 and 990 patients, respectively. During follow-up, 54 hysterectomy patients (6.04 %) and 20 sacrocolpopexy patients (2.02 %) developed a new diagnosis of opioid abuse or dependence. The opioid abuse-free survival probability at the end of the study period was significantly lower in the sacrocolpopexy group (67.4 %) compared to the hysterectomy group (83.2 %) (log-rank p < 0.0001). The hazard ratio for opioid abuse or dependence was 6.91 (95 % CI: 4.07–11.73), indicating a markedly elevated risk associated with hysterectomy.

## Discussion

Our results revealed higher postoperative risk of both opioid and antimicrobial use in the hysterectomy cohort. KM analysis showed that 25.9 % of hysterectomy patients experienced postoperative antimicrobial use compared to 14.1 % of sacrocolpopexy patients. Similarly, 44.4 % of hysterectomy patients used opioids with greater incidence of abuse or dependence compared to 15.6 % of those undergoing sacrocolpopexy. While postoperative diagnoses of opioid abuse or dependence were more common in the hysterectomy cohort (6.04 % vs. 2.02), the overall incidence remained relatively low in both groups. In contrast, no statistically significant differences were observed in postoperative antimicrobial resistance suggesting comparable rates of long-term complications from antibiotics across groups. Incidence of paralytic ileus was also addressed but did not demonstrate statistical significance.

Our intent was to compare the procedures themselves, hysterectomy and sacrocolpopexy, rather than to contrast specific surgical techniques. Although the operative route varies by institution and surgeon expertise, both operations share a common pelvic field and similar postoperative challenges related to pain control, infection prevention, and medication stewardship. Analyzing all approaches collectively allowed us to capture the overall pharmacologic and complication patterns intrinsic to each operation, providing findings that are broadly generalizable across diverse practice settings. The range of indications for hysterectomy may explain its association with higher rates of postoperative opioid use in our analysis which include conditions that may be symptomatically treated with opioids [7]. Previous studies corroborate our results demonstrating a correlation between preoperative pain, chronic pelvic pain, and endometriosis with postoperative opioid use while asserting that hysterectomy status is positively correlated with an ongoing opioid prescription [8], [9]. Pelvic organ prolapse surgery involving mesh, such as sacrocolpopexy, and hysterectomy are also associated with postoperative opioid use. However, rates of prolonged use have been shown to decrease drastically from the early postoperative period in cases of hysterectomy and procedures involving mesh [10]. This aligns closely with our findings which demonstrate increased postoperative opioid use with hysterectomy and low risk of dependence or abuse with both procedures.

Global data on surgical approaches for sacrocolpopexy and hysterectomy further clarify contributing factors to the differences in postoperative opioid and antibiotic use. Open abdominal hysterectomy comprises 64 % of hysterectomies in the United States and 80 % globally whereas sacrocolpopexy is largely performed via minimally invasive approaches [11], [12]. The open abdominal approach is associated with elongated recovery times, greater reliance on opioids for pain control, and higher risk of infectious complications compared to minimally invasive approaches [13], [14], [15]. Conversely, attenuated opioid use is correlated with shorter recovery times and reduced pain thus highlighting opportunities for improvement of patient well-being and satisfaction with minimally invasive surgeries [4], [16]. Moreover, unlike hysterectomy, sacrocolpopexy avoids vaginal cuff formation thus eliminating a direct source for infection by vaginal microbiota [17]. Therefore, widespread preference of the open abdominal approach and vaginal cuff formation with hysterectomy likely contribute to the observed higher rates of postoperative opioid and antibiotic use.

In our analysis, event-free survival was high in both cohorts, with 98.89 % of hysterectomy and 98.28 % of sacrocolpopexy patients remaining free from resistance diagnoses (log-rank p = 0.1199). Although antibiotic exposure was greater among hysterectomy patients, increased resistance did not occur, potentially reflecting appropriate perioperative prophylaxis and stewardship. Despite low resistance rates, improving practices to further reduce resistance is ideal and may be achieved with appropriate surgical planning. Of note, similarly successful antibiotic prophylaxis with administration of one dose or three doses prior to sacrocolpopexy illuminates an avenue for reducing adverse reactions and resistance [18]. This objective may be further realized by appropriate selection of a surgical approach. Unlike sacrocolpopexy, infection rates vary significantly with surgical route in hysterectomy [2], [19], [20]. With improved understanding of these postoperative outcomes, physicians can more effectively tailor care and counsel patients on postoperative expectations and risks for hysterectomy and sacrocolpopexy.

Tailored perioperative care may be achieved though enhanced recovery after surgery (ERAS) protocols which emphasize early mobilization and feeding while eliminating patient-controlled anesthesia. ERAS is also associated with reduced hospital stays, costs, and opioid use [21]. In abdominal sacrocolpopexy, ERAS with non-opioid anesthetics effectively reduces postoperative pain while maintaining outcomes [22]. Similarly, in laparoscopic hysterectomy, ERAS has been shown to reduce opioid use, with patients reporting comparable postoperative abdominal pain scores to non-ERAS groups [23]. Weston et al. supported these findings by demonstrating reduced intra-operative opioid use and postoperative pain scores by implementing ERAS with minimally invasive hysterectomy [24]. ERAS protocol may also be considered for open abdominal surgeries with reduced, but not statistically significant, post-operative opioid use and hospital stay reported with abdominal hysterectomy [25]. However, more studies are necessary to evaluate the efficacy of ERAS with open abdominal procedures. Overall, ERAS protocol is a promising approach to reduce postoperative opioid use across various surgical approaches for hysterectomy and sacrocolpopexy. ERAS also offers a valuable method to modify opioid prescribing patterns to tailor postoperative care and circumvent opioid overprescribing in pelvic surgery [26].

The clinical and surgical relevance of our study is shaped by its design. The international scope augmented generalizability, applicability, and facilitated more extensive propensity score matching. KM survival analysis enabled time-adjusted, censoring-aware comparisons between cohorts, thereby enhancing the robustness and interpretability of results. This approach accounted for differences in follow-up duration and allowed for more accurate estimation of risks related to antimicrobial use, opioid use, resistance, and abuse or dependence. Despite the advantages of using a global database, the retrospective nature of our study introduces inherent limitations. Of note, a retrospective design limited our ability to control confounders such as preoperative and intraoperative anesthetic strategies and variation in surgical techniques given multi-institutional data collection. Additionally, postoperative pain scores could not be monitored for the cases included in our study, which would provide further insight into the observed differences in opioid use. Future studies addressing pain scores and rates of infection by route of procedure for hysterectomy and sacrocolpopexy could help to further address the differences observed in our study. Additionally, prospective trials incorporating ERAS protocols into abdominal and minimally invasive methods of sacrocolpopexy and hysterectomy could help to identify strategies to reduce opioid use while maintaining optimal surgical outcomes.

## Conclusions

In this large, multicenter cohort study comparing hysterectomy and sacrocolpopexy, we observed that patients undergoing hysterectomy had a significantly higher incidence of postoperative antimicrobial and opioid use/abuse after adjusting for prior exposure. While antimicrobial resistance remained low and comparable between groups, paralytic ileus was observed only in the hysterectomy cohort. These findings underscore the need for tailored surgical planning and perioperative strategies to reduce unnecessary medication exposure and improve recovery in pelvic reconstructive surgery.

## CRediT authorship contribution statement

**Muhammed A.M. Hammad:** Conceptualization, Methodology, Formal analysis, Data curation, Writing – original draft, Visualization, Project admistaration. **MacKinnly T. Knoerzer:** Investigation, Writing – review & editing. **Gamal M. Ghoniem:** Conceptualization, Supervision, Validation, Writing – review & editing, Funding acquisition, Resources.

## Ethics statement

As a federated research network utilizing de-identified data, studies using the TriNetX network do not require institutional review board approval or informed consent.

## Declaration of Generative AI and AI-assisted technologies in the writing process

The authors did not use generative AI or AI-assisted technologies in the development of this manuscript.

## Funding

This research did not receive any specific grant from funding agencies in the public, commercial, or not-for-profit sectors.

## Declaration of Competing Interest

The authors declare that they have no known competing financial interests or personal relationships that could have appeared to influence the work reported in this paper.

## Data Availability

All data used is available on the TriNetX research network, www.trinetex.com.

## Footnotes

Appendix A Supplementary data associated with this article can be found in the online version at doi:10.1016/j.eurox.2025.100433.
