# Supplementary material for: Comparative analysis of postoperative outcomes following hysterectomy versus sacrocolpopexy: Insights from global federated health research network
Source: Eur J Obstet Gynecol Reprod Biol X. 2025 Oct 30;28:100433. doi: 10.1016/j.eurox.2025.100433 (PMC12639297; doi:10.1016/j.eurox.2025.100433)
Supplement: Supplementary file 1 — Supplementary material [file mmc1.docx]

**Supplementary Material**

**Table 1: Female genital prolapse incidence across the 993 matched patients in both groups:**

| **ICD-10 Code** | **Diagnosis** | **Hysterectomy n (%)** | **Sacrocolpopexy n (%)** | **p-value** | **SMD** |
| --- | --- | --- | --- | --- | --- |
| N81 | Female genital prolapse | 100 (10.07%) | 301 (30.31%) | <0.0001 | 0.521075 |
| N81.4 | Uterovaginal prolapse, unspecified | 39 (3.93%) | 34 (3.42%) | 0.550997 | 0.026762 |
| N81.1 | Cystocele | 36 (3.63%) | 111 (11.18%) | <0.0001 | 0.291546 |
| N81.2 | Incomplete uterovaginal prolapse | 36 (3.63%) | 92 (9.27%) | <0.0001 | 0.231192 |
| N81.6 | Rectocele | 21 (2.12%) | 106 (10.68%) | <0.0001 | 0.35535 |
| N81.3 | Complete uterovaginal prolapse | 17 (1.71%) | 32 (3.22%) | 0.030023 | 0.097493 |
| N81.9 | Female genital prolapse, unspecified | 13 (1.31%) | 10 (1.01%) | 0.52922 | 0.02824 |
| N81.5 | Vaginal enterocele | 10 (1.01%) | 27 (2.72%) | 0.004785 | 0.126866 |

Multiple varieties of POP demonstrated higher preoperative incidence among patients undergoing sacrocolpopexy compared to hysterectomy. Appropriately, POP was more frequently encountered as the indication for surgical intervention with sacrocolpopexy.

**Table 2: Compared Outcomes and their Associated Codes:**

| **Outcome** | **Associated Codes** |
| --- | --- |
| **Overall Postoperative Complications** | CPT: 1008663; ICD-10-CM: T81.4, N99.82B, I82.40, I82.409, I82.419, I82.429, I82.439, I82.489, I82.481, I82.499, I82.621, I82.622, I82.723, I82.724, I82.729, I82.89, I82.91, I82.99, T81.719A, T26, I78 |
| **Opioid Utilization** | VA: CN101; CPT: 08632, 08633; ICD-10-CM: F11.9x series (e.g., F11.90, F11.93, F11.94, F11.98) |
| **Opioid Abuse or Dependence** | ICD-10-CM: F11.1x and F11.2x series (e.g., F11.10, F11.120, F11.159, F11.20, F11.220, F11.23, F11.282); Z79.891 |
| **Antimicrobial Utilization** | VA: AM000 (includes AM114, AM200, AM250, AM300, AM400, AM700, AM900) |
| **Antimicrobial Resistance** | ICD-10-CM: Z16 |

Postoperative outcomes for all matched patients were analyzed within TriNetX. Search terms spanned five categories relevant to our study with multiple terms included per assessed outcome to ensure comprehensive analysis.
